# Investigating the Public Sentiment in Major Public Emergencies Through the Complex Networks Method: A Case Study of COVID-19 Epidemic

**DOI:** 10.3389/fpubh.2022.847161

**Published:** 2022-03-29

**Authors:** Guang Yang, Zhidan Wang, Lin Chen

**Affiliations:** School of Education Science, Jiangsu Normal University, Xuzhou, China

**Keywords:** public sentiment, major public emergency, COVID-19, complex networks, Weibo, big data

## Abstract

The main purpose of this study is to investigate what topic indicators correlate with public sentiment during “coronavirus disease 2019 (COVID-19) epidemic” and which indicators control the complex networks of the topic indicators. We obtained 68,098 Weibo, categorized them into 11 topic indicators, and grouped these indicators into three dimensions. Then, we constructed the complex networks model of Weibo's topics and examined the key indicators affecting the public's sentiment during the major public emergency. The results showed that “positive emotion” is positively correlated with “recordings of epidemic” and “foreign comparisons,” while “negative emotion” is negatively correlated with “government image,” “recordings of epidemic,” and “asking for help online.” In addition, the two vertexes of “recordings of epidemic” and “foreign comparisons” are the most important “bridges” which connect the government and the public. The “recordings of epidemic” is the main connection “hub” between the government and the media. In other words, the “recordings of epidemic” is the central topic indicator that controls the entire topic network. In conclusion, the government should publish the advance of the events through official media on time and transparent way and create a platform where everyone can speak directly to the government for advice and assistance during a major public emergency in the future.

## Introduction

Coronavirus disease 2019 (COVID-19) is a global pandemic caused by severe acute syndrome coronavirus 2 (SARS-CoV-2). This significant public crisis is characterized by outreach, sociability, sensitivity, urgency, complexity, and widespread public involvement (1). Social media has not only become an important source of information, but also given the public an open and free forum to discuss public events *via* the internet. Today, the internet has become an important medium for the general public to express their opinions and release their emotions. Netizens frequently publish information containing attitudes, viewpoints, desires, and even behavioral tendencies following major public emergencies (2).

## Investigating Public Sentiment Through Social Media

It should be noted that social media has become the main approach to investigate public sentiment. Cao et al. (3) discovered that conventional sentiment evaluation systems are based solely on textual sentiment analysis. This is limited to short texts that do not have clear sentiment words. It is difficult to determine the sentiment of a short text in microblog posts without clear sentiment words. However, identifying the pictures published by users could help analysts better understand the users' sentiment, as a result, the authors proposed a cross-media public sentiment analysis system that details the sentiment outcome by topic, region, microblog content, and user diffusion. Huang et al. (4) proposed a framework that can be used to build and improve models of public epidemic sentiment based on theories such as system dynamics and cross-validation. A specific SD model was built to simulate the dissemination process of a public sentiment system by analyzing the mechanisms and influencing factors of online public sentiment dissemination. The findings showed that increased government responsiveness promoted positive social sentiment. Positive individual behavior suppressed negative emotions while also promoting the spread of positive emotions. Zhang et al. (5) investigated the development of an “impression ecology” mobile platform for more accurately predicting public sentiment. By comparing the decision performance of long short-term memory (LSTM) and recurrent neural networks (RNNs) models on the emotional level of the public environment, the researchers verified the LSTM's performance in prediction, level decision-making effectiveness, and robustness. According to the findings of Xenos and Moy (6), premature responses to public emergencies, improper guidance to public opinions on the internet, adverse instructions to the public, and inappropriate choices of adaptation strategies for public emergencies all affect netizen's negative emotions.

## Application of The Big Data Methods

The big data method has created new stimulation for the research of public sentiment (7–10). Hu et al. (11) accrued over 300,000 geotagged tweets in the United States from March 1, 2020 to February 28, 2021 and examined the spatiotemporal patterns of public sentiment and emotion over time at the national and state levels. The researchers discovered that in most states, an increasing trend in positive sentiment was observed in conjunction with a decrease in negative sentiment, reflecting the public's rising confidence and anticipation toward vaccines. Kang et al. (12) used social media found in vaccine-related blog posts, information articles, media reports, and news reports on websites widely shared by Twitter users in the United States to build and analyze semantic networks and investigate vaccine sentiment. The key concepts of a positive emotional network related to health and medicine, and the key concepts of a negative emotional network related to organizations such as the CDC, the mainstream media, the vaccine industry, doctors, pharmaceutical companies, and the United States. Carlos de las Heras-Pedrosa et al. (13) crawled 106,261 communication samples from Twitter, YouTube, Instagram, official media websites, and internet forums. The emotional intensity of four primitive emotions, namely, anger, fear, disgust, and sadness, was measured by IBM Watson and the emotional weight of written communication was classified by Python3. The results showed that the issues related to COVID-19 pandemic include management, social cooperation, death, protection, and lack of foresight.

## Complex Networks

The complex networks which introduce complex systems theory into the analytical framework of social governance have become hot research topics in management, sociology, and psychology in the recent years (14, 15). The complex networks are neither regular networks nor random networks (16). The typical complex network consists of many vertexes and edges that connect two vertexes, where the vertexes are used to represent the variables of the system and the edges are used to represent the relationships between the variables. This can be abstracted as a graph G = (V, E) composed of the vertex set V and edge set E.

Using the SIR epidemic model and social networks theory, Lie et al. (17) analyzed the interactions among government, media, and netizens in time and space and found that 101 major incidents occurred in China between 2010 and 2017, according to empirical research conducted with China's Sina Weibo. The researchers showed that the spread of online public sentiment conforms to the scale-free and small-world characteristics, according to simulation findings. In the early stages of public sentiment development, the media's influence is insufficient; therefore, the government needs to make relevant information publicly available. The government also needs to strengthen the public's judgment on information and reduce false information. Doing so will reduce the value of information dissemination by individuals while maximizing the effectiveness of media communications. Lili et al. (18) investigated the mechanism of the dynamic evolution of online public opinion by conducting a study of dynamic interaction among government, media, and netizens from the perspective of network members and network relations. The researchers pointed out that netizens are the fundamental force behind the dynamic evolution of the public opinion network. As an information carrier to disseminate public opinion, online media has created prerequisites for the rapid dissemination of public opinion online. Media attention, press continuity, comment openness, and press bias influence the process of dynamic development of online public opinion, making the process a driving force for the dynamic evolution of such opinion. Zhang et al. (19) used the mean-field theory based on the Barabási–Albert scale-free network to update the SEIR model of infectious disease and improve the public opinion network diffusion model during the public crisis. The researchers took “COVID-19 explosion” as an example and browsed through 180 days of related microblogs, forwards, comments, and likes on Weibo. This case study illustrated that during the stable period, the propagation fluctuates in response to stimulation of events. Then, after the fluctuation, the propagation drops sharply to a relatively higher level than before and remains at a steady level again. In addition, the position of the initial disseminators is of great significance in the process of public opinion network diffusion.

## Present Study

In summary, previous studies on public emotion and complex networks mainly focused on the combination of the dynamic model and the complex networks to (1) simulate the polarization process of public emotion with the development of public opinion under the joint action of external factors and individual internal factors or (2) establish a social relationship network to simulate the interaction and communication of emotions among users. However, to date, no one has explored the relationship between the rise and fall of different public opinion topics on social media and public emotion in combination with the empirical study of big data. Therefore, the main goal of this study was to investigate what topic indicators correlate with public sentiment during COVID-19 pandemic and to model the complex networks of public topic indicators to examine which indicators play a prominent role in the entire topic network. This will help the government find out the central topic indicators of public opinion on social media to affect the development of public opinion in major emergencies, improve public emotion by controlling relevant topic indicators, prevent the continuous deterioration of public opinion in major emergencies, and promote the stability of online communities.

## Research Method

### Data Collection

**Figure 1:**
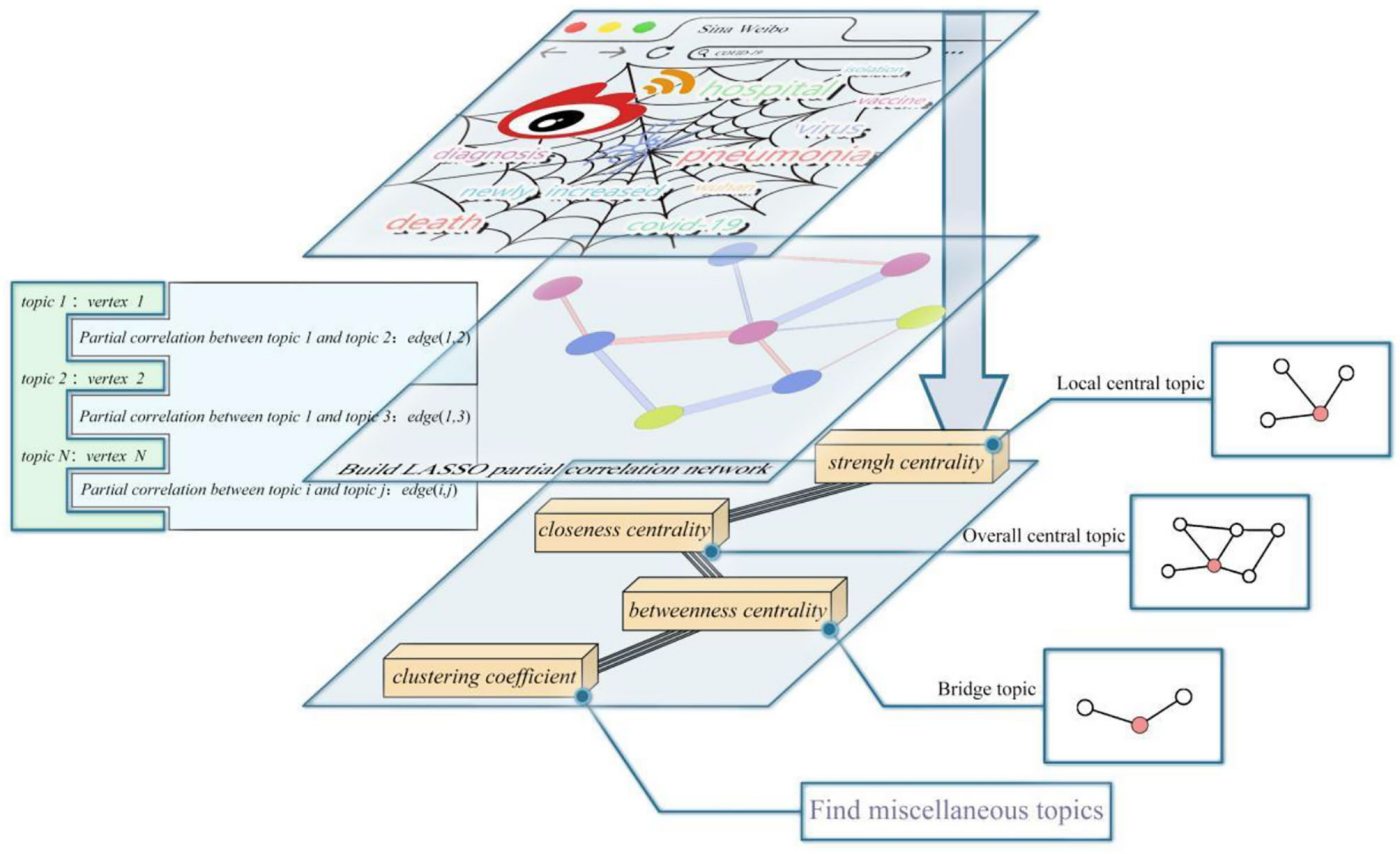
The research framework.

The investigation process is shown in Figure 1. We crawled 200,000 tweets from January 23, 2020 to February 12, 2020 and from January 6, 2021 to January 25, 2021 during the outbreak period using keywords such as “epidemic situation,” “confirmed diagnoses,” and “pneumonia” *via* Python crawler. First, the research assistants manually cleaned the data and removed duplicate contents and commercial ads, and finally, 68,098 Weibo were obtained. Second, the research assistants manually labeled the rest of the data and categorized it into 11 topics (20). Then, the 11 thematic indicators were grouped into three dimensions: government, public, and media (21). Taking 11 thematic indicators distributed among the government, the public, and the media as variables, and taking “the number of Weibo on the day of each thematic indicator/the total number of Weibo per day,” the number of Weibo in each topic for 40 days is obtained. The number of Weibo in this thematic indicator is used to describe the attention and quantity of this thematic indicator, establishing a network for studying the network characteristics of the government, the public, and the media. Specific topic indicators are given in Table 1.

**Table 1:** The key topic indicators of Weibo.

**Dimension**	**Topic indicators**	**Topic content**
Government	Epidemic prevention measures (G1)	Instructions or measures issued by the central or local government on the epidemic situation.
	Popular science answering (G2)	Experts or government agencies to answer people's doubts about the epidemic, which is scientific and authoritative.
	Scientific research (G3)	The latest scientific research achievements of scientific institutions in COVID-19.
	Refuting rumors (G4)	Official announcements and reports to break rumors.
	Government's negligence (G5)	The public or the official media accused the government of its mistakes.
	Government image (G6)	The public praised and affirmed the party and the government for epidemic control.
The public	Positive emotion (P1)	Generally, it refers to personal Weibo who still records a good life and expresses positive energy in the epidemic situation.
	Negative emotion (P2)	Generally, it refers to the difficulty and loss in the epidemic situation and records the personal Weibo of personal negative emotion.
Medium	Asking for help online (M1)	People or organizations seek help through online media.
	Recordings of epidemic (M2)	Digital broadcasting of the real-time development trend of the epidemic situation released by the media.
	Foreign comparisons (M3)	Foreign epidemics are reported in the form of news, which is in sharp contrast with domestic epidemic control.

### Statistical Analysis

A popular network model is the Pairwise Markov Random Field (PMRF) (22). PMRF is a network whose vertexes represent variables and are connected by undirected edges without arrows. The presence of a connection indicates a conditional dependency between two variables, and the line thickness represents the strength of the dependency. Furthermore, two unconnected variables are relatively independent. This model helps to clearly and explicitly interpret the edge performance parameter as the strength of the unique association between the variables.

To deal with the relatively small sampling problem, a network is constructed using “Minimum Absolute Shrinkage and Selection Operator” (23), and the adaptive least absolute shrinkage and selection operator (LASSO) networks use this regularization technique to limit the number of pseudoedges and to better obtain the interpretable network composed of new variables. The goal of adaptive LASSO is to estimate the partial correlation coefficient through the LASSO penalty (24), which leads to a weak connection that will automatically shrink to exactly zero, producing a sparse network that removes possible pseudoedges (25). Optimal adaptive LASSO networks are very sparse while maintaining a high probability. If the data do come from a sparse network with two-two interactions, this is actually the process of converging the network (26).

All the statistical analysis is performed by R software version 4.1. Given the small sample size of this study, it may not meet the standard normal distribution. If the observed variables are continuous and not normally distributed, the variables can be transformed into a normal marginal distribution. The latest network model is known as the hybrid graphical model, which also utilizes LASSO estimation and extended Bayesian information criteria (EBIC) model selection. The use of EBIC selection to perform regularized bias correlation of LASSO networks is implemented in the EBIC glasso function of the graph package of R software (26, 27). Using glasso (graphical LASSO) in conjunction with the EBIC model, a Gaussian graphical model (28) is estimated by inputting the variance-covariance matrix computed from the original data and penalizing the elements of the variance-covariance matrix to estimate the network structure by penalizing the regression coefficients in a series of multiple regression models (29). The multigrid (or multiseries and Pearson) correlation was computed using the corauto function in the qgraph package and using the R package lavaan (30). A concise network of partial correlation coefficients is eventually returned. Finally, we use a 1,000 samples bootstrap test to examine edge stability and find their correlation stability coefficients, called the CS coefficient. It quantifies the portion of the data that can be discarded, which ensures with 95% certainty that the coefficients generated by the remaining data correlate with the coefficient of the original data by at least 0.7. Ideally, this coefficient should be higher than 0.5 and should be at least higher than 0.25 (31).

### Centrality and Clustering Coefficient Analysis

The essence of the network graph vertex problem is to understand the “importance” of the vertexes in the network. This is similar to the “reins of power” held by certain actors in social networks. The purpose of the centrality measurement is to quantify these notions of “importance,” to facilitate the answers to these questions (32)—the vertexes in the network are not equally important when determining the network structure (33). The centrality index can be regarded as an operational definition that explains the importance of vertexes. The three most commonly used centrality indices are briefly described later.

Strength centrality: mainly used to quantify the degree to which a vertex is directly connected to other vertexes around it, as defined below:


$$\begin{array}{l} s_{i}=C_{D}^{w}\left(i\right)=\overset{N}{\underset{j}{\sum }}w_{ij} \end{array}$$

Where, *N* is the number of vertexes in the network and *w*_*ij*_ is the edge weight from vertex *i* to vertex *j*.

Closeness centrality: Quantifies the degree to which one vertex is indirectly connected to other vertexes. Therefore, if the distance between one point and all other points in the network is short then the point is said to have high closeness centrality, the more likely it is to be in the core of the network, so closeness centrality is an indicator that portrays the power of actors in the network, which is specifically defined as follows:


$$\begin{array}{l} C_{c}^{w\alpha }\left(i\right)={\left[\overset{N}{\underset{j}{\sum }}d^{w\alpha }\left(i,j\right)\right]}^{-1} \end{array}$$

Where, *d*^*wα*^(*i, j*) denotes the weighted shortest path from a vertex *i* to vertex *j* and α while is the tuning parameter.

Betweenness centrality: Quantifies the importance of one vertex in the average path between two other vertexes (22, 34, 35). If the betweenness centrality of a point is high, it means that the point is on many interactions network paths and this point can be considered to be in an important position. If a point' betweenness centrality is 0, it means that the point does not control any actor and is at the edge of the network. Intuitively, betweenness centrality reflects the importance of a vertex as a “bridge” and is defined as follows.


$$\begin{array}{l} C_{B}^{w\alpha }\left(i\right)=\frac{g_{jk}^{w\alpha }\left(i\right)}{g_{jk}^{w\alpha }} \end{array}$$

Where, $g_{jk}^{w\alpha }\left(i\right)$ is the number of weighted shortest paths through two vertexes, *g*_*jk*_(*i*) is the number of weighted shortest paths through a vertex *i* and α is the tuning parameter.

In addition, the clustering coefficient is also a very important metric even in complex networks, which describes the average ratio between the edges that actually exist near a vertex and the edges that could exist in complex networks (36). It shows the aggregation of a vertex in the network, that is, the aggregation of the network, that is to say, the probability that two adjacent vertexes of the same vertex are still adjacent vertexes, which reflects the local characteristics of the network. This can be quantified as an indicator of the redundancy of vertexes in the neighborhood (37). The following analysis uses Zhang and Horvath's version of the clustering coefficient (38).

It is necessary to check whether there is a relationship between the centrality index and clustering coefficient because the centrality index is often inflated by high clustering in the network (39). Many adjacent point vertexes with high clustering coefficients are interconnected. Even if the edge of the vertex is blocked, its adjacent vertexes will be interconnected directly or through a short path. If most of the vertex neighbors are interconnected, deleting the vertex will not become more difficult. The plus of low centrality indicates that this is not a key vertex in the network. Therefore, indicators with a high clustering coefficient and low centrality are often peripheral. Eliminating such topic indicators can simplify the complicated network.

## Results

### Stability of the Network

**Figure 2:**
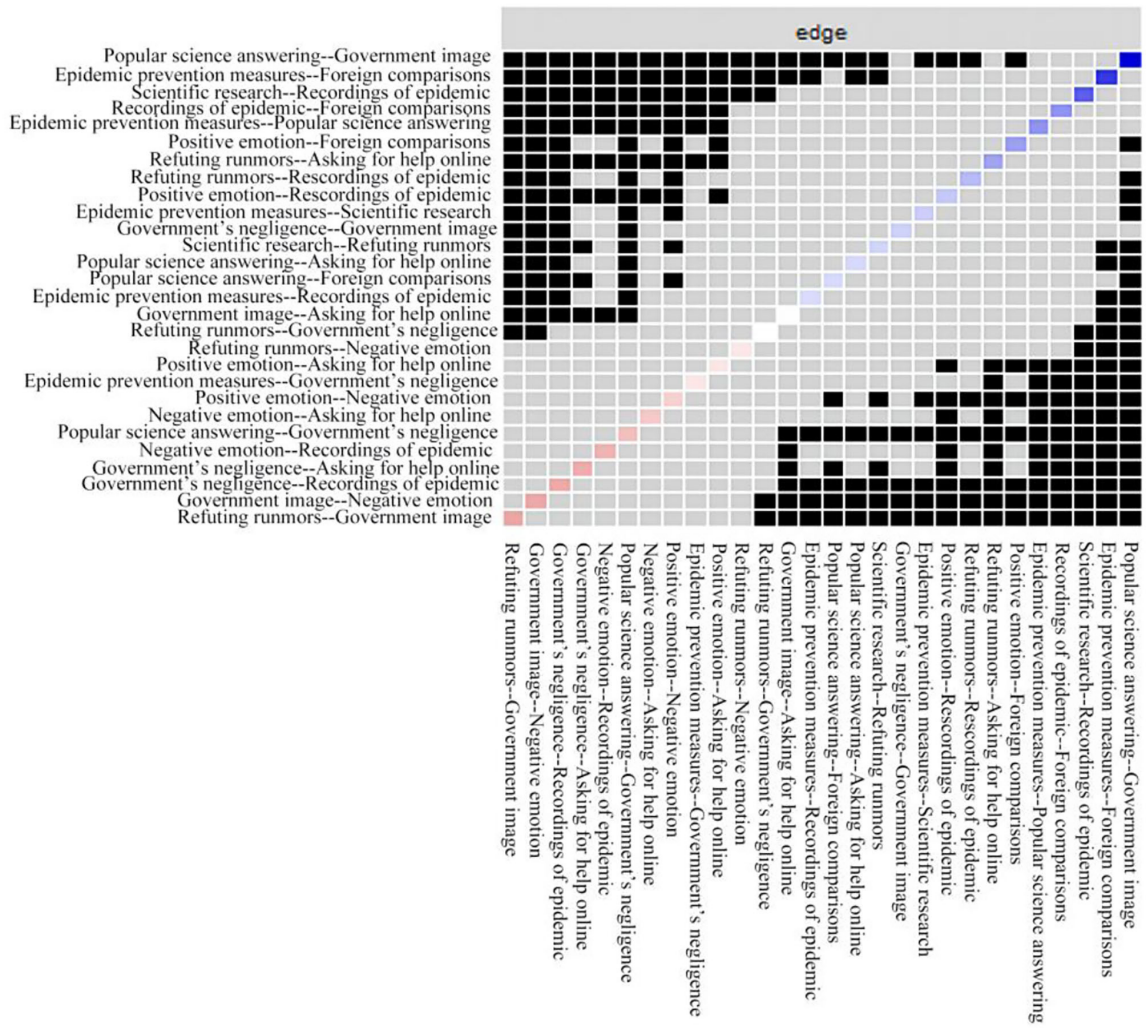
The edge weight difference test of the Weibo topic network.

The small black boxes represent statistically significant differences between two corresponding edge weights (*P* < 0.05) and small gray boxes represent statistically insignificant differences between two corresponding edge weights (*P* > 0.05). The CS coefficient of the edge of the network is 0.45 and the stability of the edge of the network meets the standard (Figure 2).

### Correlation of the Topic Indicators

**Figure 3:**
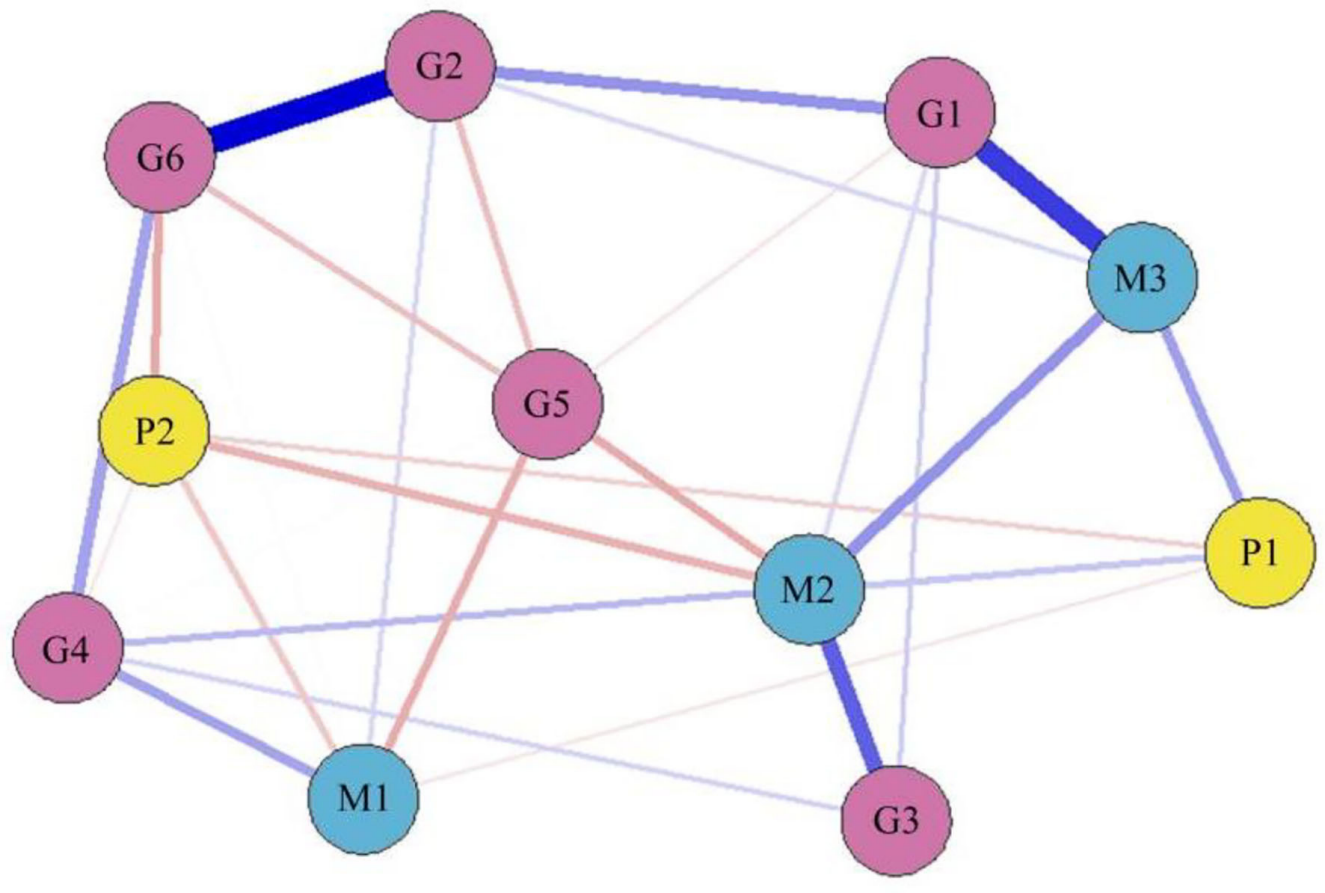
The Weibo topic network. Blue line represents positive correlation and the red line represents negative correlation. The thicker the edge, the greater the correlation between two vertexes, and the thinner the edge, the smaller the correlation between two vertexes.

The relationship between public sentiment and the topic indicators was analyzed through computing the correlations between the public's “positive emotion” and “negative emotion” and the other topic indicators. The results showed that “positive emotion” is positively correlated with “recordings of epidemic” and “foreign comparisons,” while negatively correlated with “negative emotion.” The “negative emotion” is negatively correlated with “government image,” “recordings of epidemic,” and “asking for help online” (Figure 3).

### Analysis of the Prominent Indicators

**Figure 4:**
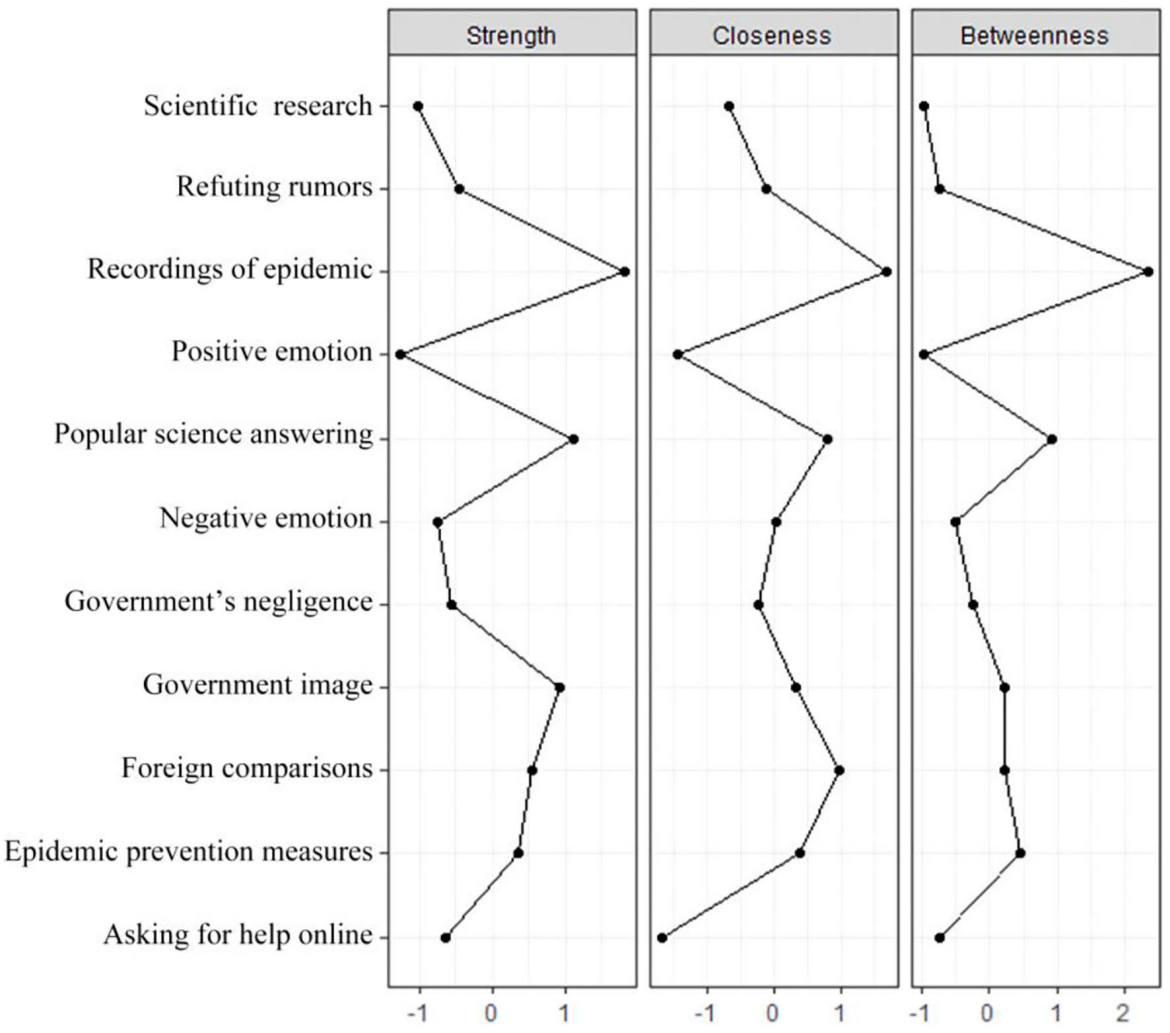
The Weibo topic network centrality Z score chart.

From Figure 4, it can be seen that the prominent indicators in intensity centrality are “recordings of epidemic” and “popular science answering,” which shows that these two indicators have a strong influence on the surrounding local network topic indicators. The prominent topic indicators close to centrality are “recordings of epidemic,” “foreign comparison,” and “popular science answering.” It shows that these three indicators are the most central indicators of the topic network, which have the most influence on topic indicators in the whole network and the strongest ability to “control” other topics. The prominent indicators in the betweenness centrality are “recordings of epidemic” and “popular science answering” which shows that these two indicators are on the path between many other two points and are the “bridge” between other topics.

**Figure 5:**
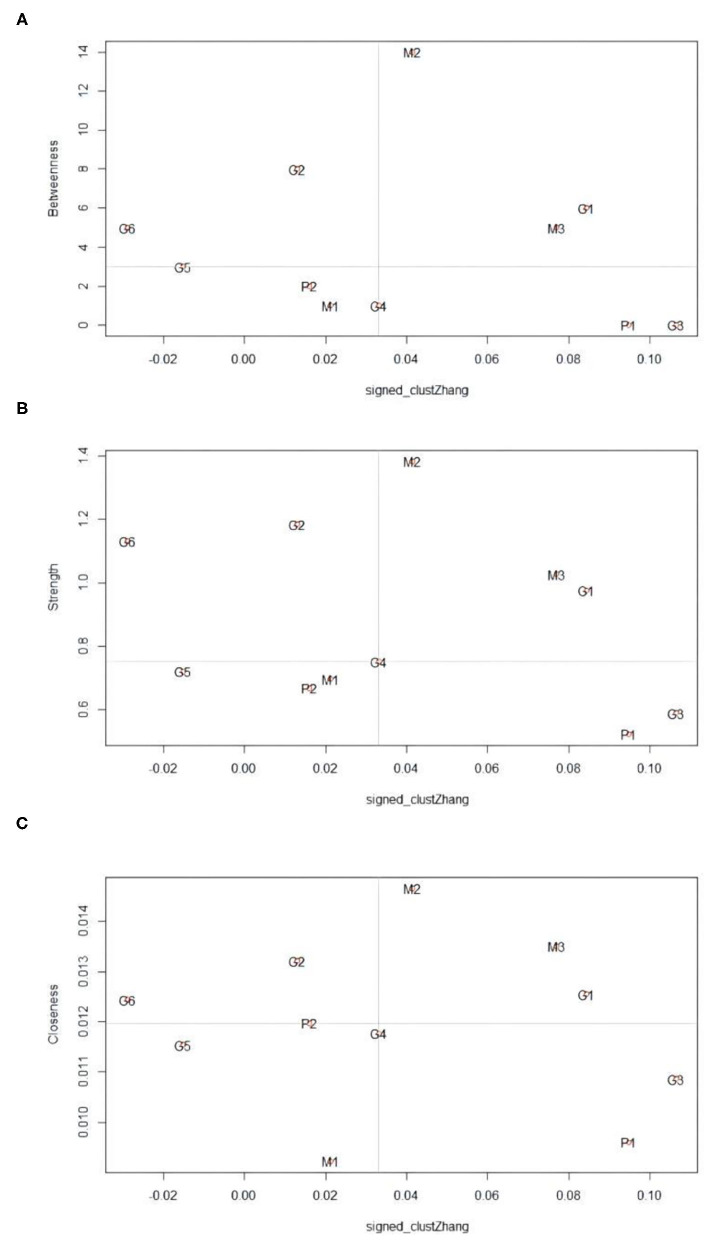
The relationship between the centrality of the topic network and the clustering coefficient in Weibo.

As shown in Figure 5, the vertexes with high clustering coefficient and low centrality in the lower right corner of the figure are “scientific research” and “positive emotion,” which shows that these two topic indicators “scientific research” and “positive emotion” have complicated overlapping effects. “Scientific research” may overlap with “popular science answering.” The “positive emotion” and “negative emotion,” which also describe public emotion, are both topical indicators reflecting public emotion, but the number of Weibo of “positive emotion” is far less than that of “negative emotion,” and its influence is not as good as that of “negative emotion.”

## Discussion

The results showed that “positive emotion” correlates with “epidemic records” and “foreign comparison,” while “negative emotion” correlates with “government image,” “asking for help online,” and “epidemic records.” It suggested that the public's positive emotions are affected mainly by the media dimension, while the public's negative emotions are affected mainly by the government and media dimensions. The two vertexes of “epidemic records” and “foreign comparison” are the main “bridges” connecting the government and the public. To sum up, public sentiment is mainly influenced by the two topical indicators of “epidemic records” and “foreign comparison.” In addition, “epidemic records” is the third most central topic indicator and is thus the main contact “hub” between the government and the media, indicating that the government uses mainly the official media to convey the epidemic situation to the public and then influences and controls the whole topic network. “Foreign comparison” as a central topic index following “epidemic records” influences public sentiment by comparing the epidemic situation with domestic and foreign epidemic prevention measures.

Therefore, in the future, it is possible to intervene with public sentiment through the following three approaches. First, the governments need to publish events transparently through the official media. According to some studies, the influence of anxiety and anger on the tendency of public opinion information dissemination in major public emergencies is regulated by expert's explanations of the scientific authority of events and accurate information release (40). Wang (41) found that timely and transparent disclosure of information by the government and the media increases public confidence, thereby enhancing confidence in epidemic prevention and control; simultaneously, the public's perception of epidemic risk decreases with the enhancement of confidence in epidemic prevention and control. Trust in the official information sources can significantly positively predict confidence and positive emotional experience. Therefore, the official media serves as the “big artery” for guiding the public in major emergencies, the government acts as the backbone of authoritative science, and high reaction speed serves as the “pulse of vitality”—lowering the public's risk perception of the major public emergencies and increasing the public's positive sentiment.

Second, governments should establish a straightforward public-oriented initiative and help-seeking platform. Zhu (42) pointed out that the appeal of interest is the core variable of network mood changes in emergencies. It is worth noting that seeking help online relates to both the positive and negative sentiments of the public. This is consistent with the findings of Carlos de las Heras-Pedrosa et al. (13). The same exchange of news, information, or media has a peak in different emotions, which results in a mixture of positive and negative emotions. This can be explained by the fact that confiding needs and dissatisfaction on the public platform is a process through which people relieve pressure; however, seeing other people's negative demands can create empathy and amplify negative emotions (43). At the same time, the public is more inclined to forward emotional microblogs, especially those with negative emotions, than to forward microblogs with neutral emotions (44). When uncontrolled, the spread of negative emotions complicates the ecological environment of network information. Hong (45) nationwide survey of more than 2,000 US citizens found a positive association between government online services and the public's trust in government. As a result, the author suggested that the government launch a citizen-government direct mode initiative and help-seeking platform that would not only make the public trust the government to alleviate the pressure but also help the government trace public problems. This would also prevent the public from spreading negative emotions by confiding in the public platform, which would detonate potential negative emotions from users and create a “public opinion vortex.”

Finally, the government should actively establish and maintain a good image to avoid dereliction of duty. If the public directly or indirectly determines that the cause of adverse results in major emergencies was the government's dereliction of duty, the government's image will be seriously affected. Therefore, in response to emergencies, the government should use the internet to conduct crisis analysis and establish a perfect emergency mechanism. If the government concerned has been absent, the public opinion of “adding fuel to the fire,” such as government dereliction of duty, will make the government fall into the “Tacitus trap.” This will result in a lack of support and isolation for the government, greatly reduce the image of the government, detonate negative public emotions, and ultimately endanger social stability. According to Figure 3, there is a strong positive correlation between government image and answers from popular science. Therefore, the government can improve its image by offering the public timely answers to popular science questions related to the event; doing so will enhance public positive sentiment during major emergencies.

### Limitation and Future Directions

This study has some limitations. The sample size is not large enough and the generalization of the proposed method requires more cases and data for testing. The selection of topic indicators may be incomplete, and some important topic indicators may be left out. A future study could attempt to establish a dynamic slicing network to investigate the differences and changing trends among netizen's emotions through these microblog topic indicators during various periods and try to establish a model to predict the time series of public emotions through the theme and number of microblog topics. Such a project could realize a new field of analyzing public sentiment from the development of public opinion.

## Conclusion

Complex networks can be used in major emergencies to identify the central vertex topics in the public opinion network and understand the relationships among them. We found that the topic indicators that affect the public's positive emotions include “epidemic records” and “foreign comparison.” Topic indicators that affect public negative emotion include “government image,” “asking for help online,” and “epidemic records.”

## Data Availability Statement

The raw data supporting the conclusions of this article will be made available by the authors, without undue reservation.

## Ethics Statement

This study was reviewed and approved by the Ethical Committee of Jiangsu Normal University.

## Author Contributions

GY and ZW conceptualized the study and wrote the manuscript. GY designed the methodology, the experiments, and performed the experiments. GY and LC analyzed the data. All authors contributed to the article and approved the submitted version.

## Funding

This study was supported by the National Social Science Foundation of China (Grant Number 20BSH096) to ZW and Jiangsu Graduates Scientific Research innovation Project (KYCX21_2517).

## Conflict of Interest

The authors declare that the research was conducted in the absence of any commercial or financial relationships that could be construed as a potential conflict of interest.

## Publisher's Note

All claims expressed in this article are solely those of the authors and do not necessarily represent those of their affiliated organizations, or those of the publisher, the editors and the reviewers. Any product that may be evaluated in this article, or claim that may be made by its manufacturer, is not guaranteed or endorsed by the publisher.
